# Three-dimensional racetrack memory devices designed from freestanding magnetic heterostructures

**DOI:** 10.1038/s41565-022-01213-1

**Published:** 2022-09-22

**Authors:** Ke Gu, Yicheng Guan, Binoy Krishna Hazra, Hakan Deniz, Andrea Migliorini, Wenjie Zhang, Stuart S. P. Parkin

**Affiliations:** Max Planck Institute of Microstructure Physics, Halle, Germany

**Keywords:** Magnetic devices, Spintronics

## Abstract

The fabrication of three-dimensional nanostructures is key to the development of next-generation nanoelectronic devices with a low device footprint. Magnetic racetrack memory encodes data in a series of magnetic domain walls that are moved by current pulses along magnetic nanowires. To date, most studies have focused on two-dimensional racetracks. Here we introduce a lift-off and transfer method to fabricate three-dimensional racetracks from freestanding magnetic heterostructures grown on a water-soluble sacrificial release layer. First, we create two-dimensional racetracks from freestanding films transferred onto sapphire substrates and show that they have nearly identical characteristics compared with the films before transfer. Second, we design three-dimensional racetracks by covering protrusions patterned on a sapphire wafer with freestanding magnetic heterostructures. We demonstrate current-induced domain-wall motion for synthetic antiferromagnetic three-dimensional racetracks with protrusions of up to 900 nm in height. Freestanding magnetic layers, as demonstrated here, may enable future spintronic devices with high packing density and low energy consumption.

## Abstract (web summary)

A lift-off and transfer method enables the fabrication of efficient three-dimensional racetrack memory devices fabricated from freestanding magnetic heterostructures on a prepatterned substrate and may—in the future—allow for advanced three-dimensional nanostructures in next-generation nanoelectronic devices with a low device footprint.

## Main

Magnetic random access memory is a leading candidate for the realization of next-generation memory devices due to its high performance and non-volatility^1–3^. Although magnetic random access memory stores individual bits in magnetic tunnel junction devices, magnetic racetrack memory (RTM) encodes data in a series of magnetic domain walls (DWs) that are manipulated by electric current pulses within a single racetrack element^4,5^. Thus, RTM goes beyond magnetic random access memory and has the potential to realize vastly greater data capacities with much higher speeds. Early RTM devices have evolved from in-plane magnetized^6^ to out-of-plane magnetized^7,8^ two-dimensional (2D) racetracks into, most recently, synthetic antiferromagnetic (SAF) 2D racetracks^9^ in which two magnetic sublayers are antiferromagnetically coupled through a thin ruthenium layer. The pursuit of higher-data-density three-dimensional (3D) devices would make RTM even more attractive^10–12^. However, conventional methods to deposit the required thin-film structures on pre-formed 3D structures, or exfoliation of 2D magnetic materials and dry transfer onto 3D structures, are difficult to implement^13,14^. In addition, the Curie temperatures of exfoliable van der Waals ferromagnetic (FM) materials are typically far below room temperature, for example, Fe_3_GeTe_2_ (~205 K)^15^ and Cr_2_Ge_2_Te_6_ (<61 K)^16^.

Recently, a lift-off and transfer technique utilizing a water-soluble material Sr_3_Al_2_O_6_ (SAO) has been applied to form freestanding layers of various perovskite-based oxides, including FM La_0.7_Sr_0.3_MnO_3_ (ref. ^17^), ferroelectric BaTiO_3_ (ref. ^18^) and superconducting YBa_2_Cu_3_O_7–*x*_ (ref. ^19^), even down to the monolayer limit^20^. Here by using such a freestanding film transfer technique, we show that complex heavy-metal (HM)/FM heterostructures deposited onto an SAO layer formed on SrTiO_3_ (STO) can be transferred onto a given substrate (a sapphire substrate here), with the magnetic properties largely intact. We also illustrate Hall-bar and RTM devices that are fabricated from the freestanding films and demonstrate that they exhibit comparable performance with the as-deposited films. We further realize a 3D RTM device by transferring the magnetic thin-film heterostructures onto a sapphire substrate on which 3D protrusions of different heights were pre-formed. We observe the current-induced motion of DWs in these 3D structures. We systematically examine how the current-induced DW behaviour depends on the protrusion height and geometry. We show that chiral DWs of different types can be selectively passed across the protrusions by controlling the angle between the racetrack and protrusion. Thus, we show that racetracks formed on 3D protrusions have substantial potential as key components in DW logic devices^21,22^. We also realized efficient 3D RTM devices utilizing freestanding SAF films with protrusions up to ~900 nm high that show similar DW velocity versus current density curves to the 2D films.

## Lift-off and transfer of HM/FM heterostructures

**Fig. 1:**
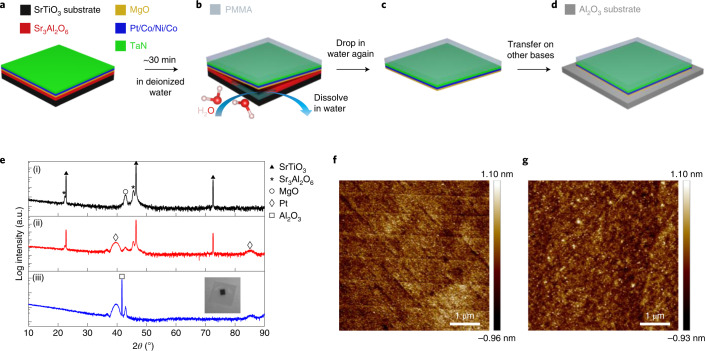
Formation of freestanding multilayer (Pt/Co/Ni/Co) and evaluation of the transfer process. **a**, Deposition of an SAO/MgO/Pt/Co/Ni/Co epitaxial multilayer on an STO(001) substrate and capped with TaN. **b**, Immersion of the as-deposited film in deionized water to dissolve the SAO layer after spin coating PMMA. **c**, Separation of a freestanding multilayer (MgO/Pt/Co/Ni/Co/TaN). **d**, Transfer of the freestanding multilayer onto a sapphire substrate. **e**, Out-of-plane *θ*–2*θ* XRD patterns of SAO/MgO (i), SAO/MgO/Pt/Co/Ni/Co/TaN (ii) and a freestanding multilayer transferred onto a sapphire substrate (iii). The inset in (iii) shows a freestanding multilayer on a 10 × 10 mm^2^ sapphire substrate. **f**,**g**, AFM images of the as-deposited sample (**f**) and a freestanding multilayer (**g**) transferred onto a sapphire substrate. The root mean square roughness values in **f** and **g** are 0.278 nm and 0.280 nm, respectively.

**Extended Data Fig. 1:**
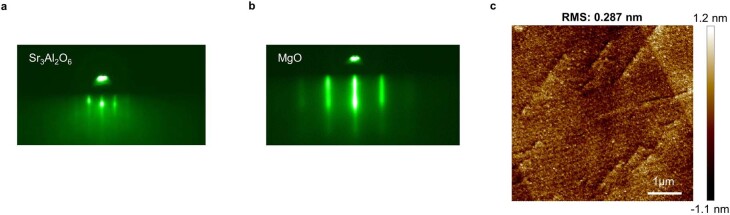
Deposition of Sr_3_Al_2_O_6_ (SAO)/MgO bilayer thin films. **a**,**b**, In-situ RHEED patterns of SAO (**a**) and SAO/MgO (**b**) deposited on SrTiO3 (001) in a PLD chamber. The preservation of the high surface quality can be seen in the line-type RHEED patterns taken at the end of SAO and MgO growth. **c**, AFM image of as-deposited SAO/MgO. A smooth surface with a root mean square roughness equal to 0.287 nm was obtained.

**Extended Data Fig. 2:**
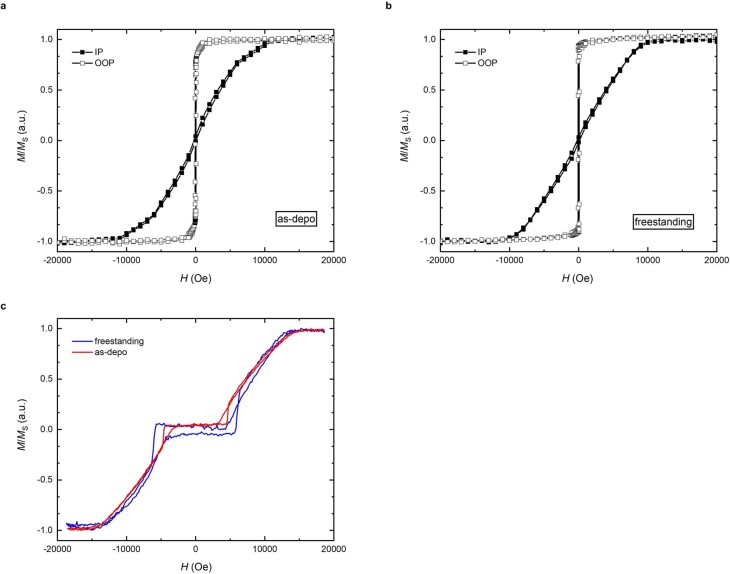
Magnetic property of as-deposited and transferred samples. **a**,**b**, In-plane (IP, filled square) and out-of-plane (OOP, open square) magnetization (*M*)/saturated magnetization (*M*_s_) versus field (*H*) curves of as-deposited HM/FM sample (**a**) and freestanding HM/FM sample (**b**) measured by SQUID at room temperature. *M*_s_ is the average magnetization at high field. **c**, Out-of-plane magnetization (*M*)/saturated magnetization (*M*_s_) versus field (*H*) curves of as-deposited SAF sample (red) and freestanding SAF sample (blue) measured by MOKE at room temperature.

We deposited HM/FM heterostructures formed from Pt(50)/Co(3)/Ni(7)/Co(3) with a TaN(30) capping layer by magnetron sputtering onto an SAO(200)/MgO(100) bilayer (all units in Å), which are hereafter called the as-deposited samples. The SAO/MgO bilayer is prepared via pulsed laser deposition on an STO(001) substrate (Fig. 1a and Extended Data Fig. 1). After the heterostructure is prepared, a thin poly(methyl methacrylate) (PMMA) protection layer is spin coated onto the film. Then, the sample is immersed in deionized water to remove the SAO sacrificial release layer so that the magnetic thin-film stack is separated from the STO substrate and can be transferred to a sapphire substrate, hereafter called the freestanding sample (Fig. 1b–d). Methods details the growth and transfer process. We then compare the X-ray diffraction (XRD) patterns of SAO/MgO deposited on an STO(001) substrate without any magnetic heterostructure (Fig. 1e(i)), with the as-deposited sample (Fig. 1e(ii)) and freestanding sample (Fig. 1e(iii)). A diffraction peak originating from the face-centred cubic(111)-oriented Pt layer can be clearly seen in the as-deposited sample as well as after the transfer process: this guarantees a strong out-of-plane magnetization of both as-deposited and freestanding samples. Our method allows for freestanding samples with large areas of more than 3 × 3 mm^2^ (Fig. 1e(iii), inset), which facilitates subsequent device fabrication and measurements. From the analysis of atomic force microscopy (AFM) images (Fig. 1f,g), the root mean square roughness of the as-deposited and freestanding samples are found to be nearly identical, indicating very smooth films with small roughness both before and after the transfer process. All the film characterization methods demonstrate no degradation of the film quality in the transfer process, which corresponds well with the magnetization measurements (Table 1 and Extended Data Fig. 2a,b).

**Table 1:** Comparison of magnetic properties of as-deposited thin films and freestanding samples

	*H*_DMI_ (Oe)	*H*_C_ (Oe)	*H*_K_ (Oe)	*M*_S_ (emu cm^−3^)	${{{\boldsymbol{K}}}}_{{{\mathbf{u}}}}^{{{{\mathbf{eff}}}}}$ (erg cm^−3^)	*Δ* (nm)	*D* (erg cm^−2^)
As-deposited	1,059.08 ± 65.96	64.86	8,260	820	3.39 × 10^6^	5.43	0.47 ± 0.03
Freestanding	1,007.86 ± 28.59	77.43	8,604	840	3.61 × 10^6^	5.26	0.44 ± 0.01

To calculate the DMI constant, an exchange stiffness constant (*A*) of 1.0 μerg cm^−1^ was used.

## DW motion in freestanding HM/FM heterostructures

**Fig. 2:**
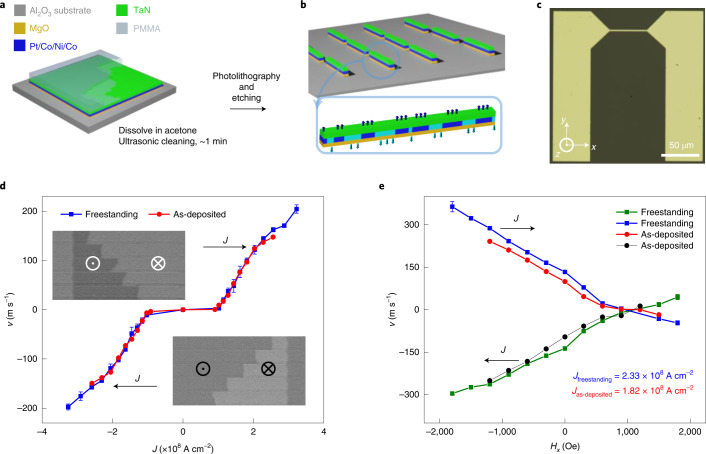
CIDWM in freestanding racetrack (Co/Ni/Co). **a**, Protective PMMA is removed. **b**, Schematic of device fabrication from a freestanding Pt/Co/Ni/Co heterostructure. The cyan and blue colours correspond to down- and up-magnetized domains, respectively. **c**, Optical image of a typical racetrack. The device comprises a nanowire (length, 50 μm; width, 3 μm) and two bond pads. DW motion and current direction are along the *x* axis. **d**,**e**, Current-induced DW velocity in freestanding Co/Ni/Co transferred onto a sapphire substrate (blue and olive; square) and as-deposited samples (red and black; circle) without (**d**) and with (**e**) a magnetic field applied along the *x* axis. To examine the longitudinal-field dependence of the CIDWM, a fixed current density of 2.33 × 10^8^ A cm^−2^ for the freestanding racetrack and 1.82 × 10^8^ A cm^−2^ for the as-deposited sample are used. The insets in **d** show typical Kerr images of DW motion in response to a series of injected 10-ns-long current pulses (*J* = 2 × 10^8^ A cm^−2^) composed of three pulses in freestanding multilayers transferred onto a sapphire substrate. The bright and dark regions correspond to down (⊗ or ↓) and up (⊙ or ↑) domains, respectively. The error bars in **d** and **e** represent the standard deviation.

After transferring the exfoliated films onto a sapphire substrate, RTM devices are then fabricated using standard photolithography techniques and Ar-ion milling (Fig. 2a,b). A straight wire that is 50 µm long and 3 µm wide is fabricated (Fig. 2c). Current-induced domain-wall motion (CIDWM) in this racetrack is then measured in both as-deposited and freestanding samples with Kerr microscopy. The DW velocity of both as-deposited and freestanding samples is plotted against the injected current density (Fig. 2d). Interestingly, almost identical CIDWM behaviours are observed before and after the transfer process, with the only difference in the high-current-density region where the thermal nucleation of multi-domains takes place more readily in the as-deposited samples compared with the freestanding samples. This is due to the greater thermal conductivity through the sapphire substrate (Methods). The longitudinal-field dependence of the CIDWM at a given current density (*J* = 1.82 × 10^8^ A cm^−2^ for the as-deposited sample and *J* = 2.33 × 10^8^ A cm^−2^ for the freestanding sample) is also examined in both samples (Fig. 2e) and the Dzyaloshinskii–Moriya interaction (DMI) effective field *H*_DMI_ is determined by the linear fitting of these curves^23,24^. The DMI constant *D* of both samples are calculated using the saturation magnetization *M*_S_ and effective magnetic anisotropy constant $K_{\mathrm{u}}^{{{{\mathrm{eff}}}}}$ obtained from magnetization measurements by applying in-plane and out-of-plane magnetic fields (Extended Data Fig. 2a,b and Methods)^23^. The magnetic properties, including the DMI effective field *H*_DMI_, coercive field *H*_C_, effective uniaxial anisotropy field *H*_K_, saturation magnetization *M*_S_, effective uniaxial magnetic anisotropy constant $K_{\mathrm{u}}^{{{{\mathrm{eff}}}}}$, DW width *Δ* and DMI constant *D* of both samples are summarized in Table 1. All these magnetic properties show similar values in the two samples, which indicates that the film quality was preserved through the transfer process and, in turn, results in almost identical CIDWM in both samples.

## Transport properties of HM/FM heterostructures

**Fig. 3:**
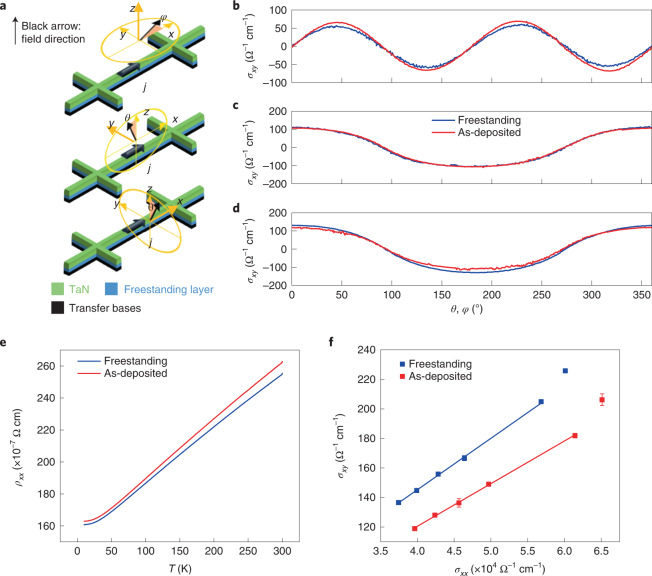
Transport properties of freestanding Pt/Co/Ni/Co. **a**, Schematic of the transport measurement geometry. **b**–**d**, Hall conductivity (*σ*_*xy*_) in freestanding and as-deposited samples as a function of azimuthal and polar angles (*φ*, *θ*) defined in **a**. **e**,**f**, Longitudinal resistivity (*ρ*_*xx*_) as a function of temperature (*T*) (**e**) and Hall conductivity (*σ*_*xy*_) versus longitudinal conductivity (*σ*_*xx*_) (**f**) of the freestanding and as-deposited samples. The blue and red lines in **f** are linear fits of *σ*_*xy*_ versus *σ*_*xx*_ for the freestanding and as-deposited samples, respectively. In these two cases, the fitted line has a slope that is equal to 0.0035 ± 0.0001 (freestanding sample) and 0.0029 ± 0.0001 (as-deposited sample). The slope is the physical coefficient related to the extrinsic contribution of the skew scattering mechanism. The low-temperature (10 K) data are excluded from the fits.

**Extended Data Fig. 3:**
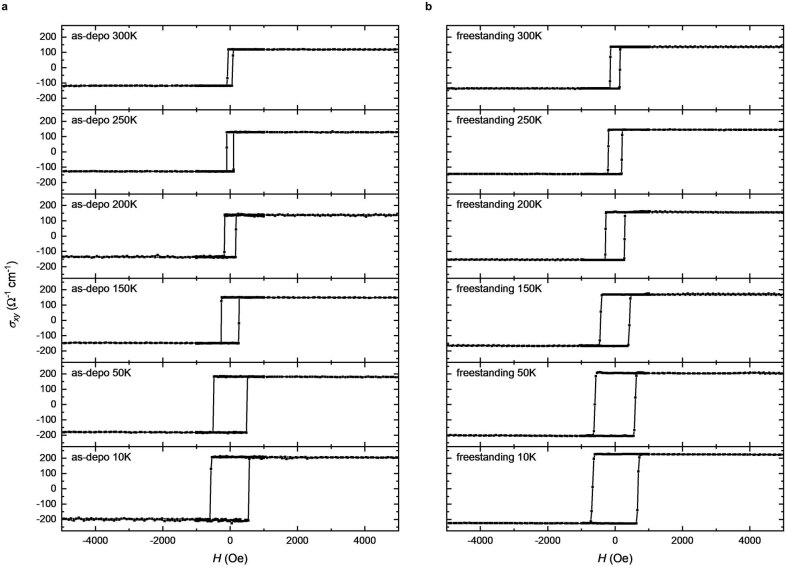
Hall conductivity (*σ*_*xy*_) of as-deposited and freestanding HM/FM samples at various temperatures. **a**,**b**, *σ*_*xy*_ versus *H*, shown from top to bottom, measured at 300 K, 250 K, 200 K, 150 K, 50 K and 10 K of as-deposited (**a**) and freestanding samples (**b**).

We further pattern the as-deposited and freestanding HM/FM heterostructures into Hall-bar devices to measure the transport properties. The Hall conductance is measured with current injected along the *x* axis, whereas the applied magnetic field is rotated in the *x*–*y*, *x*–*z* and *y*–*z* planes (Fig. 3b–d), as depicted in Fig. 3a. The dependence of Hall conductivity *σ*_*xy*_ on the applied field shows almost identical behaviour in the two samples, in good agreement with the CIDWM and magnetic-property measurements. A slight difference is observed in the longitudinal resistivity *ρ*_*xx*_ as the temperature is varied (Fig. 3e). The hysteresis loop of *σ*_*xy*_ at various temperatures is summarized in Extended Data Fig. 3. When Hall conductivity *σ*_*xy*_ is plotted versus longitudinal conductivity *σ*_*xx*_ (Fig. 3f), a linear dependence that is a typical characteristic of the extrinsic origin of the Hall signal is found^25^. In the high-value region of *σ*_*xx*_ at low temperatures, a deviation from a linear fit is found, as the intrinsic contribution of the Hall conductance begins to dominate^26^.

## Three-dimensional racetracks formed from freestanding HM/FM heterostructures

**Fig. 4:**
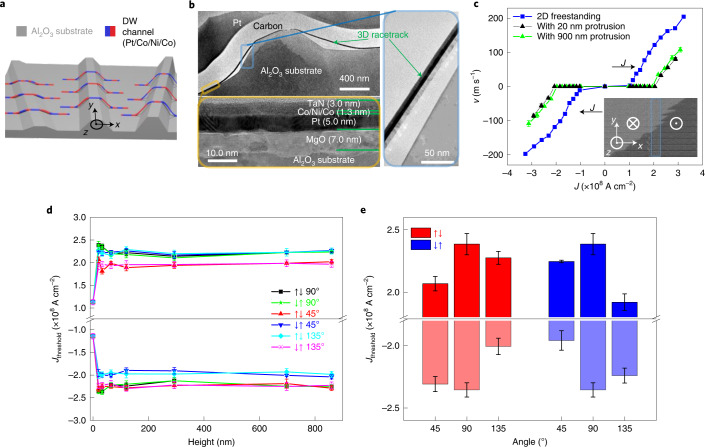
CIDWM across 3D protrusions in HM/FM heterostructures. **a**, Schematic of freestanding racetracks formed from HM/FM heterostructures transferred onto a pretreated sapphire substrate. The 3D protrusions with different heights and different angles from the DW motion direction are made on the substrate by etching. The angle is defined as the angle between the protrusion and *x* axis. The blue and red parts of the racetrack correspond to down- and up-magnetized domains, respectively. **b**, Cross-sectional TEM image of a freestanding racetrack on a 3D protrusion with a height of 700 nm and width of 3 μm (top left). Magnified high-resolution TEM images showing individual layers in the 3D racetrack of the two regions highlighted in the top-left image as an orange rectangle (bottom left) and blue rectangle (right). The horizontal green lines in the bottom-left panel indicate the interfaces between the individual layers. **c**, DW velocity versus current density in racetracks formed from the 2D freestanding films without protrusions (blue square) and with protrusions perpendicular to the racetrack channel with heights of 20 nm (black triangle) and 900 nm (green triangle). The insets show the typical Kerr images of DW motion in response to a series of injected 10-ns-long current pulses (*J* = −2.3 × 10^8^ A cm^−2^) composed of ten pulses for the 3D RTM formed on a protrusion with a height of 20 nm and angle of 90°. The bright and dark regions correspond to down (⊗ or ↓) and up (⊙ or ↑) domains, respectively. The tall blue box indicates the position of protrusion. **d**,**e**, Threshold current density required to drive a DW across 3D protrusions with various heights (0, 20, 30, 60, 120, 300, 700 and 900 nm) (**d**) and a height of 20 nm (**e**). Two types of DW (↑↓ or up–down and ↓↑ or down–up) cross the 3D protrusions at three different angles (45°, 90° and 135°) in **d**: the ↑↓ DWs cross the protrusions at an angle of 90° (black square), 45° (red triangle) and 135° (cyan diamond); the ↓↑ DWs cross the protrusions at an angle of 90° (green five-pointed star), 45° (blue inverted triangle) and 135° (magenta asterisk). The red and blue bars in **e** correspond to up–down (↑↓) and down–up (↓↑) DWs, respectively. The positive and negative values are defined with respect to the +*x* and –*x* direction, respectively. The error bars in **d** and **e** represent the standard deviation.

**Extended Data Fig. 4:**
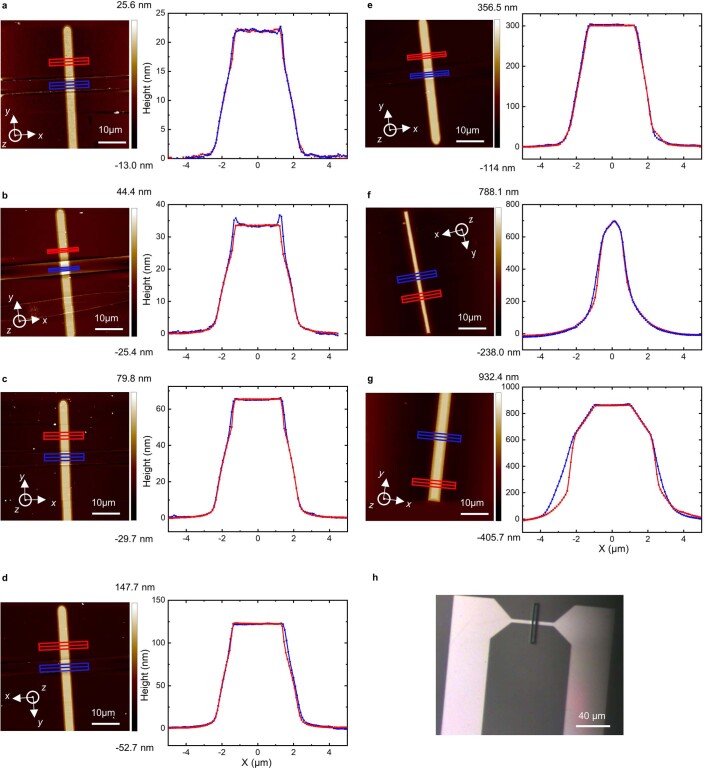
AFM images of 3D protrusions and corresponding line scans of rectangularly marked region. **a**-**g**, Left: AFM images of 3D protrusions with heights of 20 nm (**a**), 30 nm (**b**), 60 nm (**c**), 120 nm (**d**), 300 nm (**e**), 700 nm (**f**), 900 nm (**g**); Right: Corresponding line scans of rectangularly marked region (red: 3D protrusions, blue: 3D racetrack). The origin is set at the center of the rectangle and *x*-axis is perpendicular to the 3D protrusion. **h**, Optical image of a freestanding racetrack on a 3D protrusion with a height of 900 nm, width of 3 μm and angle of 90°.

**Extended Data Fig. 5:**
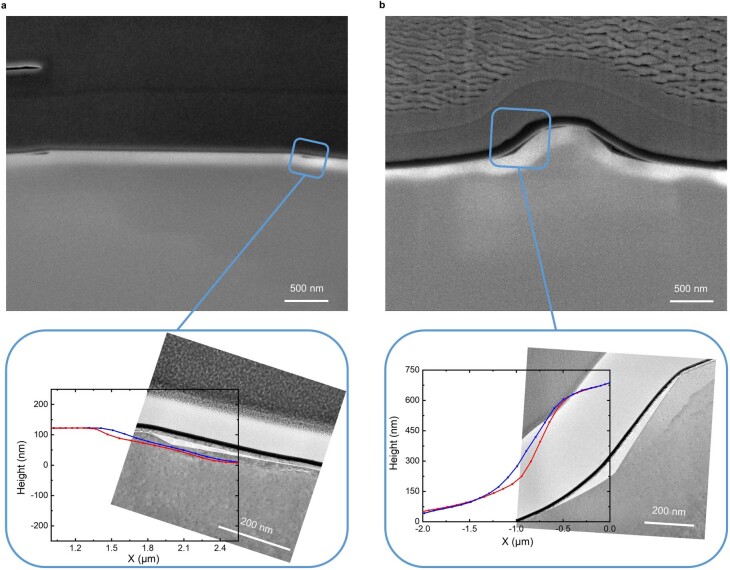
Cross-sectional SEM and TEM images of 3D racetrack devices formed from HM/FM heterostructures. Upper panel: cross-sectional SEM images of the 3D racetrack devices with protrusion heights of 120 nm (**a**) and 700 nm (**b**); Lower panel: magnified TEM image of the side region and corresponding line scans from the AFM images. Note that the difference between the line scan and the TEM image in the *x*-direction is caused by the AFM tip. This is especially evident when the tip passes the high 3D protrusion.

Having demonstrated that the performance of both racetrack and Hall-bar devices shows no obvious degradation between the as-deposited and freestanding films, we further transfer the magnetic thin-film heterostructures onto a pretreated sapphire substrate with 3D protrusions on it. The 3D protrusions are pre-etched plates with a width of 3 µm, height varying from 20 to 900 nm (Extended Data Fig. 4) and length of 50 µm. After the film is transferred to the pretreated sapphire substrate, the RTM devices are fabricated with racetracks across the 3D protrusions formed at an angle of 45°, 90° and 135°. By carrying out cross-sectional transmission electron microscopy (TEM) of the RTM devices formed on the protrusions with a height of 700 nm (Fig. 4b), the freestanding film is found to closely follow the local geometry with slight deviations, thereby forming a 3D RTM device (Fig. 4a). Extended Data Figs. 4 and 5 show other protrusion heights and the corresponding AFM, TEM and scanning electron microscopy measurement results. A slight deviation of the freestanding films from 3D protrusions is observed for higher protrusions, which may be a result of surface roughness. We note that the roughness of the surface of protrusions could be improved with other methods of fabricating these protrusions, for example, chemical etching^27^.

After the above verification of the 3D structure, we further examine the effects of 3D protrusion on the CIDWM. As shown in Fig. 4c, the efficiency of CIDWM degrades when the 3D protrusions are implemented. However, surprisingly, this degradation shows nearly no dependence on the protrusion height. Furthermore, we focus on the threshold current density *J*_threshold_ required for a DW to go across the 3D protrusion, which is a critical criterion for the realization of 3D RTM devices. Interestingly, *J*_threshold_ increases once the 3D protrusion is introduced, but remains almost constant with increasing protrusion height varying from the nanometre to micrometre scale (Fig. 4d). Here *J*_threshold_ takes a maximum value when the 3D protrusion and racetrack channel are perpendicular to each other, identical for up–down and down–up DWs in both current injection directions.

**Extended Data Fig. 6:**
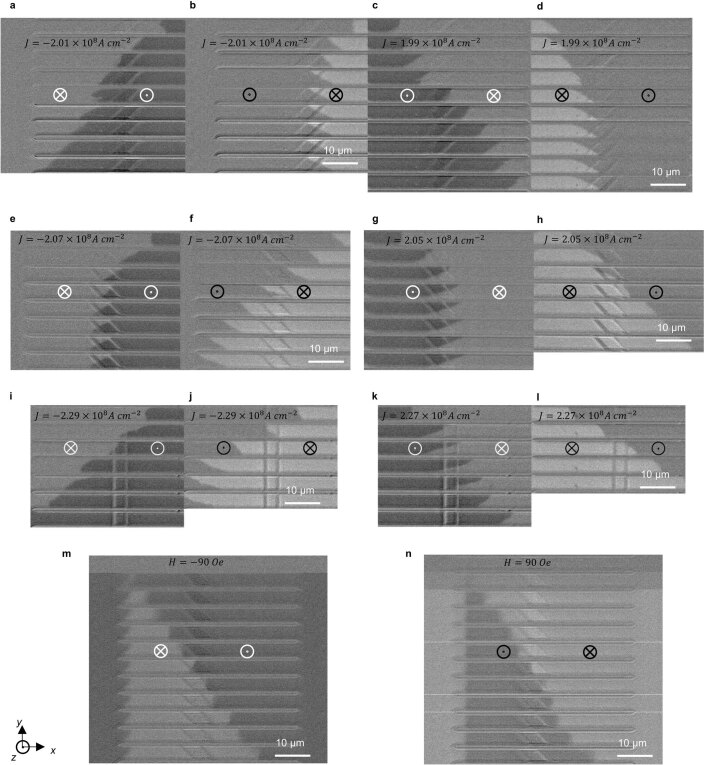
Typical Kerr images of the current and field induced domain wall (DW) motion across 3D protrusions in HM/FM racetracks. **a**-**l**, DW motion in response to a series of injected current pulses (*J*≈2 × 10^8^A cm^−2^) composed of ten × 10 ns long pulses in freestanding HM/FM racetracks across 3D protrusions with an angle of 45° (**a**-**d**), 135° (**e**-**h**) and 90° (**i-l**) with respect to the *x*-axis. **m**,**n**, DW motion across HM/FM 3D protrusions at an angle of 135° with respect to the *x*-axis in response to an out-of-plane magnetic field *H*=−90Oe (**m**) and *H*=90Oe (**n**). Each photo is taken at one-second intervals. The motion of two different types of DWs (down-up or ⊗-⊙ or ↓↑ and up-down or ⊙-⊗ or ↑↓) driven at the same current density (same direction) and same magnetic field intensity (opposite directions) are compared. The bright and dark regions correspond to down (⊗ or ↓) and up (⊙ or ↑) domains. Positive and negative values are defined with respect to *+x/+z* and *–x/-z* direction, respectively.

Moreover, *J*_threshold_ shows a very strong dependence on the angle between the 3D protrusion and racetrack channel, with respect to the DW type and injected current direction. When the racetrack is no longer formed perpendicular to the protrusion but rather at an oblique angle of 45° (135°), a different scenario occurs: for positive currents, *J*_threshold_ is distinctly smaller (greater) for an up–down DW compared with a down–up DW and vice versa for negative currents (Fig. 4e). Notably, we observe a distinct tilting of the DWs in the CIDWM of the 3D RTM devices for different DW types (Extended Data Fig. 6a–l), whereas no such tilting behaviour is observed in the field-driven case (Extended Data Fig. 6m,n). Such a non-reciprocal *J*_threshold_ results from the chirality of the DW since the DMI effective field is influenced by the protrusion geometry and shows a smaller energy barrier for a certain combination of DW type and protrusion tilting angle^28–31^. The selective passing of DWs is, thus, realized from the difference in *J*_threshold_. For example, as shown in Extended Data Fig. 6c,d, for an angle of 45° and current density of 2 × 10^8^ A cm^−2^, only the up–down DW can propagate through the protrusion, whereas the down–up domain cannot. A 3D DW ‘diode’ based on the DW type combined with the DW propagation direction is, thus, realized.

## Three-dimensional racetracks formed from SAF films

**Fig. 5:**
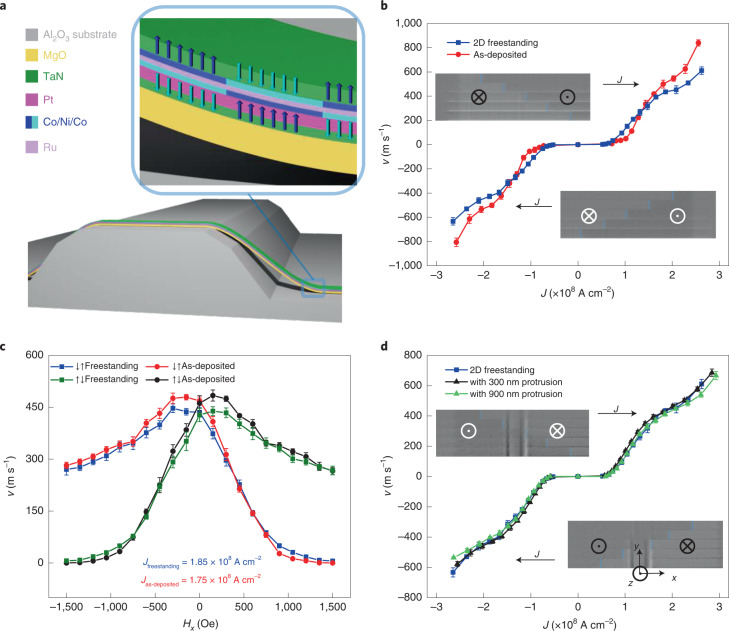
CIDWM across 3D protrusions in SAF structures. **a**, Schematic of the freestanding racetracks formed from SAF structures transferred onto a prepatterned sapphire substrate. **b**,**c**, Current-induced DW velocity in freestanding SAF films transferred onto a sapphire substrate (blue and olive; square) and as-deposited samples (red and black; circle) without (**b**) and with (**c**) a magnetic field applied along the *x* axis. To examine the longitudinal-field dependence of CIDWM, a fixed current density of 1.85 × 10^8^ A cm^−2^ for the freestanding racetrack and 1.75 × 10^8^ A cm^−2^ for the as-deposited sample are used. **d**, DW velocity versus current density in racetracks formed from the 2D freestanding films without any protrusions (blue square) and with protrusions perpendicular to the racetrack channel with heights of 300 nm (black triangle) and 900 nm (green triangle). The insets in **b** and **d** show the typical Kerr images of the DW motion in response to a series of injected 10-ns-long current pulses (*J* = 2.28 × 10^8^ A cm^−2^) composed of one pulse in the as-deposited thin films (**b**) and 10-ns-long current pulses (*J* = 1.42 × 10^8^ A cm^−2^) composed of two pulses in freestanding SAF films transferred onto a sapphire substrate with 300-nm-high protrusions and protrusion angle of 90° (**d**). The bright and dark regions correspond to down (⊗ or ↓) and up (⊙ or ↑) domains, respectively. The blue lines highlight the DW position. The error bars in **b**–**d** represent the standard deviation.

We further extend the above freestanding technique to both 2D and 3D RTM devices fabricated from SAF systems, where the CIDWM is much more efficient than in the HM/FM heterostructures^9^. The SAF films that are composed of TaN(20.0)/Pt(30.0)/Co(3.0)/Ni(7.0)/Co(1.5)/Ru(9.5)/Co(3.0)/Ni(7.0)/Co(3.0)/TaN(30.0) are deposited by magnetron sputtering onto an SAO(200.0)/MgO(100.0) bilayer (all units in Å). The same transfer method as in the HM/FM case discussed above is used (Fig. 5a). The magnetization versus magnetic-field (*M*–*H*) loops of the freestanding SAF film show almost identical behaviour with those of the as-deposited films (Extended Data Fig. 2c), which, in turn, gives rise to a similar CIDWM behaviour in both DW velocity versus current density and the response to an external longitudinal field before and after the transfer process (Fig. 5b,c). A DW velocity of up to 600 m s^−1^ is achieved in the freestanding SAF films. It is noteworthy that the longitudinal-field dependence of the current-induced DW velocity for the SAF case is extremely sensitive to variations in the magnetic parameters^9,32^. Our results, especially the similar response of DW velocity to the longitudinal magnetic field before and after transfer, again confirm the robustness of the transfer process.

Unlike the HM/FM case, where the CIDWM efficiency significantly degrades with a much lower DW velocity and a two times larger threshold current density when 3D protrusions are introduced, the 3D RTM devices formed with SAF films show almost unchanged behaviour. Almost identical CIDWM behaviours are observed in the RTM devices formed from freestanding SAF films with or without 3D protrusions, regardless of the protrusion heights (Fig. 5d): the DW can freely pass through the 3D protrusions even at ultralow DW velocities near the threshold current density. The highly efficient CIDWM in the SAF structure is very well maintained. When further varying the angle between the protrusions and racetrack channel, no degradation of the CIDWM efficiency can be observed (Supplementary Videos 1–3). For the 2D case, earlier studies have demonstrated the robustness of the RTM devices formed from the SAF structures against changes in local geometry^21,28^. Here our results show that this robustness even extends to 3D geometries. This makes the SAF system an ideal breeding ground for future highly efficient 3D RTM devices.

## Conclusions

In this work, we have successfully utilized a lift-off and transfer technique to form freestanding magnetic thin-film heterostructures on various substrates. We have demonstrated that the magnetic properties are maintained in the freestanding samples after the transfer process with comparable device performance compared with devices fabricated from the as-deposited films themselves. A 3D RTM device is realized by combining the transfer technique with traditional 2D device fabrication procedures. When utilizing freestanding SAF racetrack structures to create 3D RTM devices, we find that they exhibit similar CIDWM efficiencies as those in comparable 2D RTM devices. SAF racetracks are key to the technological development of RTM due to their high efficiency, elimination of stray magnetic fields and insensitivity to changes in local geometry. The demonstration of this concept in the third dimension is an important milestone in the further development of racetrack technologies. Our results open up a new route for racetrack fabrication, which can be applied in building 3D structures as well as in the construction of flexible devices and in the combination of exotic materials^18,33^. Next steps include taking advantage of the third dimension to facilitate the incorporation of novel writing and reading elements.

## Methods

### Sample preparation and device fabrication

SAO/MgO bilayer films were fabricated on thermally annealed STO(001) substrates using pulsed laser deposition in a vacuum system with a base pressure of 2 × 10^−8^ torr and equipped with an in situ reflection high-energy electron diffraction and a KrF excimer laser (248 nm) source. The SAO and MgO layers have thicknesses of 20 and 10 nm, respectively. The oxygen partial pressure and laser pulse frequency were 2 × 10^−6^ torr and 2 Hz, respectively, for both SAO and MgO, whereas the laser fluence and temperature were 0.7 J cm^−2^ and 750 °C for SAO and 1.7 J cm^−2^ and 700 °C for MgO, respectively. After deposition, the bilayer film was cooled down to room temperature at a maximum rate of 10 °C min^−1^ in 200 torr oxygen. After transfer into an ultrahigh-vacuum sputter deposition chamber, the SAO/MgO bilayer was annealed at 600 °C for 30 min and then Pt(50.0)/Co(3.0)/Ni(7.0)/Co(3.0)/TaN(30.0) and TaN(20.0)/Pt(30.0)/Co(3.0)/Ni(7.0)/Co(1.5)/Ru(9.5)/Co(3.0)/Ni(7.0)/Co(3.0)/TaN(30.0) were deposited using magnetron sputtering at room temperature (all units in Å).

For device fabrication, a 100-nm-thick PMMA layer was spin coated onto the as-deposited sample. The entire structure was then immersed in deionized water for ~30 min to remove the SAO layer: the multilayer structure remained on the substrate. By dipping in water for the second time, the multilayer structure was separated from the substrate. The freestanding sample together with water was then picked up and transferred onto another substrate^34^.

For the freestanding samples, racetrack nanowires (3 μm wide and 50 μm long) and Hall-bar structures with a channel width of 20 μm were fabricated using photolithography and Ar-ion milling after the 100 nm PMMA layer was removed by ultrasonic cleaning with acetone. As a control experiment, the as-deposited sample was protected with a 100-nm-thick PMMA layer formed by spin coating: this layer was removed by oxygen plasma after exposure and development.

The 3D structures were created by photolithography and subsequent Ar-ion milling at an angle of 100° from the sapphire substrate. For more complex 3D structures, more sophisticated lithographic and etching methods may be needed. The sapphire substrates with arrays of 3D protrusions were then cleaned in acetone at 50 °C using ultrasonic cleaning for 1 h to completely remove any residual polymer resist. Afterwards, the 3D racetracks were fabricated with similar parameters as those used in the 2D case (except for a small defocus of ~0.6 μm during exposure in our Heidelberg MLA 150 maskless optical lithography tool). The ion milling, as for the 2D case, was carried out an angle of 90°.

### Film characterization

The crystal structures of the films and freestanding samples were determined by high-resolution XRD using Cu Kα_1_ radiation (Bruker, D8 DISCOVER). The surface morphology was characterized using AFM (Bruker, Dimension Icon-PT AFM). Optical imaging of the device was carried out with an optical microscope with a 20× objective lens (Zeiss, Axiotron). The transverse and longitudinal resistivities of the Hall devices were measured by a four-probe method using a physical property measurement system (Quantum Design) coupled with a lock-in amplifier (AMETEK, 7270 DSP). The *M*–*H* curves were measured using a superconducting quantum interference device (Quantum Design) magnetometer at room temperature.

The DW velocities were determined from Kerr microscopy measurements using injected current pulses that had a temporal length of 10 ns. The threshold current to drive a DW across the 3D protrusions was determined by varying the magnitude of the injected current. Since, in the 3D racetrack devices the DW velocity may vary along the racetrack due to the presence of protrusions, we define the DW velocity in this case by dividing the total length of the channel with the total current pulse time used to drive the DW from one end of the channel across the protrusion to the other. When the DW is stuck within the protrusion region and thus cannot reach the other end of the channel, the total DW velocity is defined as zero. The DW is defined as being pinned by a 3D protrusion when the DW cannot be driven by a sequence of 60 10-ns-long current pulses. The threshold current driving a DW in the absence of any protrusion was measured on the same wafer in a region without any 3D protrusions.

All given standard deviations come from multiple measurements. To determine the threshold current that drives a DW across one type of 3D protrusion (that is, a 3D protrusion with a given angle and height), five different devices were characterized.

### Calculation of *D*

Here *M*_S_ and *H*_C_ of the as-deposited and freestanding samples were determined from the out-of-plane *M*–*H* curves, whereas $H_\mathrm{K}^{{{{\mathrm{eff}}}}}$ was determined from the in-plane *M*–*H* curves. Also, $K_\mathrm{u}^{{{{\mathrm{eff}}}}}$ was calculated from the expression $K_\mathrm{u}^{{{{\mathrm{eff}}}}} = \frac{1}{2}\mu _0M_{{{\mathrm{S}}}}H_\mathrm{K}^{{{{\mathrm{eff}}}}}$. The DW width was calculated using $\varDelta = \sqrt {A/K_\mathrm{u}^{{{{\mathrm{eff}}}}}}$, with exchange stiffness *A* = 1.0 μerg cm^−1^ (ref. ^32^). The DMI constant was then determined from *D* = Δ*μ*_0_*H*_DMI_*M*_S_, where *H*_DMI_ was determined from the longitudinal-field dependence of the DW velocity.

### TEM specimen preparation and investigation

The cross-section lamellae of the 3D racetrack specimens were prepared by a focused-ion-beam method (FEI, 600 Nova NanoLab DualBeam microscope). After depositing a protective bilayer (carbon/Pt) on the area of interest (approximately 10–20 × 3 μm^2^), trenches were created with an ion beam at a voltage of 30 kV on both sides of the long edges. Afterwards, the lamella was transferred to a TEM grid using a micro-transfer system. Finally, a lamella with a thickness of a few tens of nanometres was obtained by thinning and polishing. The scanning electron microscopy images were taken at a voltage of 5 kV with the same dual-beam microscope. The lamellae were then characterized by TEM (JEOL, JEM-F200) equipped with a Schottky-type electron source at an accelerating voltage of 200 kV.

## Online content

Any methods, additional references, Nature Research reporting summaries, source data, extended data, supplementary information, acknowledgements, peer review information; details of author contributions and competing interests; and statements of data and code availability are available at 10.1038/s41565-022-01213-1.

## Supplementary information

Supplementary Video 1 Kerr microscopy measurements of the DW dynamics for an SAF structure in response to a series of injected 10-ns-long current pulses (50 pulses; *J* = 0.64 × 10^8^ A cm^−2^) for a freestanding film that was transferred onto a sapphire substrate with 300 nm protrusions and protrusion angle of 90°.

Supplementary Video 2 Kerr microscopy measurements of the DW dynamics for an SAF structure in response to a series of injected 10-ns-long current pulses (90 pulses; *J* = 0.61 × 10^8^ A cm^−2^) for a freestanding SAF film that was transferred onto a sapphire substrate with 300 nm protrusions and protrusion angle of 45°.

Supplementary Video 3 Kerr microscopy measurements of the DW dynamics for an SAF structure in response to a series of injected 10-ns-long current pulses (60 pulses; *J* = 0.66 × 10^8^ A cm^−2^) for a freestanding SAF film that was transferred onto a sapphire substrate with 900 nm protrusions and protrusion angle of 90°.

## Data Availability

The data that support the findings of this study are available from the corresponding authors upon request.

## References

[1] Wolf SA (2001). Spintronics: a spin-based electronics vision for the future. Science.

[2] Parkin SSP (2004). Giant tunnelling magnetoresistance at room temperature with MgO (100) tunnel barriers. Nat. Mater..

[3] Ikeda S (2010). A perpendicular-anisotropy CoFeB–MgO magnetic tunnel junction. Nat. Mater..

[4] Parkin SSP, Hayashi M, Thomas L (2008). Magnetic domain-wall racetrack memory. Science.

[5] Parkin S, Yang S-H (2015). Memory on the racetrack. Nat. Nanotechnol..

[6] Hayashi M, Thomas L, Moriya R, Rettner C, Parkin SSP (2008). Current-controlled magnetic domain-wall nanowire shift register. Science.

[7] Miron IM (2011). Fast current-induced domain-wall motion controlled by the Rashba effect. Nat. Mater..

[8] Ryu K-S, Yang S-H, Thomas L, Parkin SSP (2014). Chiral spin torque arising from proximity-induced magnetization. Nat. Commun..

[9] Yang S-H, Ryu K-S, Parkin SSP (2015). Domain-wall velocities of up to 750 m s^−1^ driven by exchange-coupling torque in synthetic antiferromagnets. Nat. Nanotechnol..

[10] Mönch I (2011). Rolled-up magnetic sensor: nanomembrane architecture for in-flow detection of magnetic objects. ACS Nano.

[11] Schöbitz M (2019). Fast domain wall motion governed by topology and Œrsted fields in cylindrical magnetic nanowires. Phys. Rev. Lett..

[12] Sanz-Hernández D (2017). Fabrication, detection, and operation of a three-dimensional nanomagnetic conduit. ACS Nano.

[13] Maurenbrecher H (2018). Chiral anisotropic magnetoresistance of ferromagnetic helices. Appl. Phys. Lett..

[14] Castellanos-Gomez A (2014). Deterministic transfer of two-dimensional materials by all-dry viscoelastic stamping. 2D Mater..

[15] Deng Y (2018). Gate-tunable room-temperature ferromagnetism in two-dimensional Fe_3_GeTe_2_. Nature.

[16] Gong C (2017). Discovery of intrinsic ferromagnetism in two-dimensional van der Waals crystals. Nature.

[17] Lu D (2016). Synthesis of freestanding single-crystal perovskite films and heterostructures by etching of sacrificial water-soluble layers. Nat. Mater..

[18] Dong G (2019). Super-elastic ferroelectric single-crystal membrane with continuous electric dipole rotation. Science.

[19] Chen Z (2019). Freestanding crystalline YBa_2_Cu_3_O_7−*x*_ heterostructure membranes. Phys. Rev. Mater..

[20] Ji D (2019). Freestanding crystalline oxide perovskites down to the monolayer limit. Nature.

[21] Guan Y (2021). Ionitronic manipulation of current-induced domain wall motion in synthetic antiferromagnets. Nat. Commun..

[22] Zhao L (2020). Current-driven magnetic domain-wall logic. Nature.

[23] Thiaville A, Rohart S, Jué É, Cros V, Fert A (2012). Dynamics of Dzyaloshinskii domain walls in ultrathin magnetic films. Europhys. Lett..

[24] Ryu K-S, Thomas L, Yang S-H, Parkin SSP (2013). Chiral spin torque at magnetic domain walls. Nat. Nanotechnol..

[25] Hoffmann A (2013). Spin Hall effects in metals. IEEE Trans. Magn..

[26] Tian Y, Ye L, Jin X (2009). Proper scaling of the anomalous Hall effect. Phys. Rev. Lett..

[27] Berenschot JW, Tas NR, Jansen HV, Elwenspoek M (2009). Chemically anisotropic single-crystalline silicon nanotetrahedra. Nanotechnology.

[28] Garg C, Yang SH, Phung T, Pushp A, Parkin SSP (2017). Dramatic influence of curvature of nanowire on chiral domain wall velocity. Sci. Adv..

[29] Sheka DD (2022). Fundamentals of curvilinear ferromagnetism: statics and dynamics of geometrically curved wires and narrow ribbons. Small.

[30] Ryu K-S, Thomas L, Yang S-H, Parkin SSP (2012). Current induced tilting of domain walls in high velocity motion along perpendicularly magnetized micron-sized Co/Ni/Co racetracks. Appl. Phys. Expr..

[31] Boulle O (2013). Domain wall tilting in the presence of the Dzyaloshinskii-Moriya interaction in out-of-plane magnetized magnetic nanotracks. Phys. Rev. Lett..

[32] Yang S-H, Garg C, Parkin SSP (2019). Chiral exchange drag and chirality oscillations in synthetic antiferromagnets. Nat. Phys..

[33] Kum H-S (2020). Heterogeneous integration of single-crystalline complex-oxide membranes. Nature.

[34] Gu K (2020). Simple method to obtain large‐size single‐crystalline oxide sheets. Adv. Func. Mater..

